# Endoscopic ultrasound-guided hepaticogastrostomy using a biopsy needle may improve the technical success rate of the one-step technique

**DOI:** 10.1055/a-2436-6911

**Published:** 2024-10-25

**Authors:** Takeshi Ogura, Yuki Uba, Nobuhiro Hattori, Kimi Bessho, Hiroki Nishikawa

**Affiliations:** 1 Endoscopy Center, Osaka Medical and Pharmaceutical University Hospital, Takatsuki, Japan; 2 2nd Department of Internal Medicine, Osaka Medical and Pharmaceutical University, Takatsuki, Japan

Bile peritonitis is a frequent adverse event of endoscopic ultrasound-guided hepaticogastrostomy (EUS-HGS) 1 . To prevent this adverse event, a one-step technique that skips the tract dilation step has recently been reported 2 . To further improve the technical success of the one-step technique, EUS-guided biliary drainage using fine-needle biopsy (FNB) needle has been reported 3 . However, although this technique might be technically feasible with the transduodenal approach, in EUS-HGS, intrahepatic bile duct puncture might be challenging because the penetration ability of the FNB needle is poor compared with a fine-needle aspiration (FNA) needle. Recently, a flexible FNB needle with a sharper tip has become available (Sono Tip TopGain; Medi-Globe GmbH, Rohrdorf, Germany) Fig. 1 ). Compared with an FNA needle, the puncture hole might be larger with the novel FNB needle. In addition, the penetration function might be stronger than that of the conventional FNB needle 4 . Therefore, the good puncture ability and larger hole obtained with this novel needle might increase the utilization of the one-step EUS-HGS technique. EUS-HGS using the novel FNB needle is described below.

**Fig. 1:**
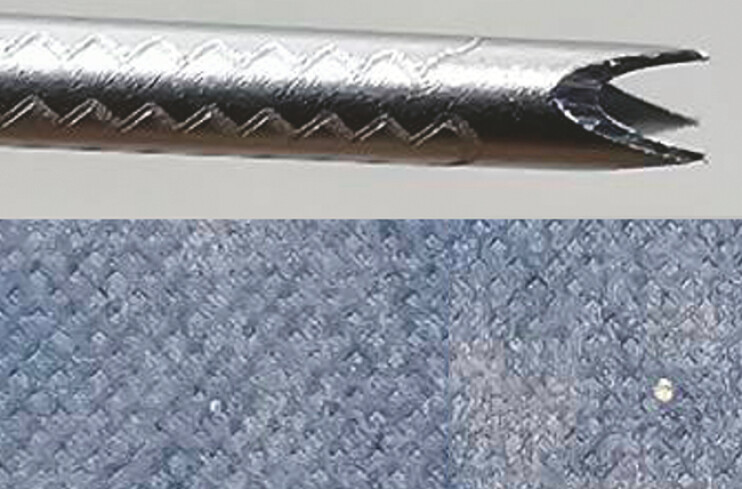
A flexible fine-needle biopsy (FNB) needle with a sharper tip (Sono Tip TopGain; Medi-Globe GmbH, Rohrdorf, Germany) (upper image). Lower images: compared with the hole made by a fine-needle aspiration needle (left), the hole is larger with the FNB needle (right).

A 77-year-old woman was admitted to our hospital with obstructive jaundice caused by advanced pancreatic head cancer. Endoscopic retrograde cholangiopancreatography failed due to duodenal obstruction, and therefore EUS-HGS was attempted. The diameter of the intrahepatic bile duct was only 1.8 mm, but because the novel 19-G FNB needle is extremely sharp, intrahepatic bile duct puncture was successfully performed. However, respiratory movement was strong and as a result, the needle penetrated the hepatic parenchyma. This needle is flexible compared with conventional FNB needles, and we could therefore easily change the axis of the needle ( Fig. 2 **a** ). After successful bile duct puncture, a 0.025-inch guidewire was inserted into the biliary tract. Although the guidewire was initially advanced into the periphery of the bile duct ( Fig. 2 **b** ), its position could be easily adjusted for correct placement because the FNB needle has a fork-tip shape allowing manipulation of the guidewire without shearing ( Fig. 2 **c** ). After successful guidewire deployment, a stent delivery system (5.9-Fr delivery system, HANARO Benefit; M.I. Tech., Seoul, South Korea) was successfully inserted without tract dilation ( Fig. 3 ). Finally, EUS-HGS was successfully performed without any adverse events ( Video 1 ).

**Fig. 2:**
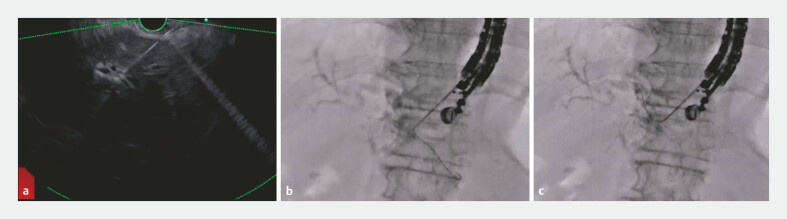
Puncture and guidewire placement. **a** Intrahepatic bile duct puncture was successfully performed. **b** The guidewire was advanced into the periphery of the bile duct. **c** The fork-tip shape of the fine-needle biopsy needle allowed easy manipulation of the guidewire without shearing.

**Fig. 3:**
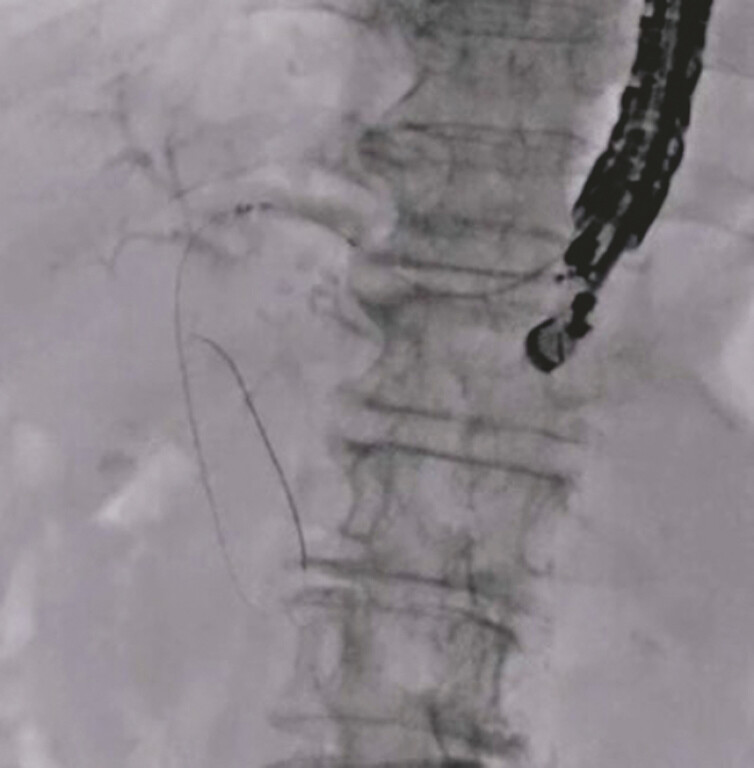
Stent delivery system (5.9-Fr delivery system, HANARO Benefit; M.I. Tech., Seoul, South Korea) was successfully inserted without tract dilation.

Endoscopic ultrasound-guided hepaticogastrostomy with the one-step technique was performed using the novel biopsy needle.Video 1

In conclusion, a novel FNB needle might facilitate the one-step technique during EUS-HGS. Further cases are required to confirm the utility of this technique.

Endoscopy_UCTN_Code_TTT_1AS_2AH

## Notes

Endoscopy E-Videos https://eref.thieme.de/e-videos
*E-Videos* is an open access online section of the journal *Endoscopy* , reporting on interesting cases and new techniques in gastroenterological endoscopy. All papers include a high-quality video and are published with a Creative Commons CC-BY license. Endoscopy E-Videos qualify for HINARI discounts and waivers and eligibility is automatically checked during the submission process. We grant 100% waivers to articles whose corresponding authors are based in Group A countries and 50% waivers to those who are based in Group B countries as classified by Research4Life (see: https://www.research4life.org/access/eligibility/ ). This section has its own submission website at https://mc.manuscriptcentral.com/e-videos .
